# Evaluation of TCR amplification in tumor lesions and peripheral blood of renal cell carcinoma patients

**DOI:** 10.1186/2051-1426-1-S1-P254

**Published:** 2013-11-07

**Authors:** Chiara Massa, Harlan Robins, Cindy Desmarais, Dagmar Riemann, Corinna Fahldieck, Paolo Fornara, Barbara Seliger

**Affiliations:** 1 Institute of Medical Immunology, Martin Luther University Halle-Wittenberg, Halle (Saale), Germany; 2 Adaptive Biotechnologies Corp, Seattle, WA, USA; 3 Clinic of Urology, Martin Luther University Halle-Wittenberg, Halle (Saale), Germany

## 

A major requirement for cancer immunotherapy is the development of biomarkers for prognosis, as well as for monitoring the success of the undergoing therapy.

In an attempt to evaluate the immune response of renal cell carcinoma (RCC) patients, tumor lesions and / or blood samples from 12 patients with clear cell RCC underwent deep T cell receptor (TCR) sequencing using the immunoSEQ assay (Adaptive Biotechnologies). Despite the low number of samples, different TCR distribution patterns could be detected. Most of the RCC patients presented "patient-specific" TCR sequences corresponding to >1% of their TCR repertoire that were shared between peripheral blood and tumor tissue. Frequently, an enrichment of these TCR sequences was found in the RCC lesion when compared to the peripheral blood of the patient. In addition, TCR patterns shared by different RCC patients were identified, which were only present in their tumor lesions, but not in peripheral blood. In particular, a group of 6 RCC patients shared between 4 and 6 TCR sequences, while other 3 RCC patients shared between 4 and 8 different TCRs. The relative frequencies of those shared TCRs among the different donors were very different, varying from below 1% to a maximum of 37% of the total TCR repertoire.

Analysis of additional samples and correlation of the TCR sequencing data with clinical parameters could lead to a correlation of particular TCRs with an RCC patient's prognosis or T cell clone expansion under therapy and thus provide important information for patient treatment.

